# Pregnancy outcomes following maternal treatment with rituximab prior to or during pregnancy: a case series

**DOI:** 10.1093/rap/rkaa074

**Published:** 2021-01-04

**Authors:** Kirstie Perrotta, Elizabeth Kiernan, Gretchen Bandoli, Rachel Manaster, Christina Chambers

**Affiliations:** Department of Pediatrics, University of California, San Diego (UCSD), La Jolla, CA, USA

**Keywords:** rituximab, pregnancy, birth defects, rheumatoid arthritis, multiple sclerosis, B cell depletion

## Abstract

**Objective:**

Rituximab is a CD20-directed cytolytic antibody used for non-Hodgkin lymphoma, chronic lymphocytic leukaemia and RA, and off label for JIA, multiple sclerosis and lupus. Owing to concerns about infant B cell depletion, the manufacturer recommends avoidance of rituximab throughout pregnancy and for 12 months before conception. The aim of this study was to add to the limited data on pregnancy outcomes in women with exposure to rituximab.

**Methods:**

Data were obtained from MotherToBaby Pregnancy Studies. Participants were enrolled prospectively into this observational study between 2007 and 2019. Pregnancy exposure and outcome data were collected from medical records, telephone interviews and dysmorphology examinations. The outcomes examined included spontaneous abortion, stillbirth, premature delivery, pregnancy complications, major and minor anomalies, small for gestational age, neonatal complications and serious infections.

**Results:**

We classified 19 women with exposure to rituximab into three groups. Group A included three women who received rituximab during pregnancy. Group B included three women who received their last infusion before conception but had assumed pregnancy exposure owing to the long half-life of the drug. Group C included 13 women who used rituximab in the 2 years before pregnancy, with the last infusion given no sooner than five half-lives before conception. Three children had a major structural defect. Preterm delivery occurred in two pregnancies, and two infants were small for gestational age on birth weight. No cases of B cell depletion were reported.

**Conclusion:**

No pattern of major structural anomalies or other adverse outcomes was reported in this case series.

**Key messages**

- Rituximab use during pregnancy has raised concern for potential immune system effects in neonates.
- No pattern of major structural anomalies or serious infections was reported in this case series.
- Clinicians should assess lymphocyte subset levels (T, B and NK cells) for newborns with rituximab exposure.

## Introduction

Rituximab is a chimeric murine/human-engineered IgG1 monoclonal antibody that depletes B lymphocytes by selectively depleting CD20-expressing cells in peripheral blood and lymphoid tissues. First approved by the US Food and Drug Administration in 1997, rituximab is used primarily to treat non-Hodgkin lymphoma, chronic lymphocytic leukaemia, RA, haematological diseases, and granulomatosis with polyangiitis. It has also been used off label to treat JIA, SLE and multiple sclerosis (MS).

Rituximab has a long elimination half-life, averaging 18–22 days as measured in non-pregnant adults with RA (range 5.17–77.5 days) [1]. Nearly complete elimination should be achieved within 3–4 months after dosing, but has been detected in some patients as long as 24 weeks after the last infusion. Transplacental transfer has been demonstrated with cord blood at term, but because rituximab contains an IgG1 component, placental transfer is expected to be negligible during the first trimester [2]. Transfer is more likely to occur beginning at 16 weeks of gestation, similar to other IgG immunoglobulins [3], and fetal blood levels may reach levels similar to maternal levels by 26 weeks of gestation. Given that rituximab can cause B cell depletion in adults and has been measured in cord blood, there is theoretical concern for depletion of fetal/infant B lymphocytes and increased risk for neonatal infection.

Despite fairly reassuring human and animal pregnancy safety data accumulated thus far, it is recommended by the manufacturer that effective contraception be used for 12 months after receiving the last infusion of rituximab before attempting pregnancy [4].

In order to add evidence to the limited available literature, we sought to describe outcomes from a small case series of pregnancies with preconception and prenatal exposure to rituximab.

## Methods

### Source of the sample

Data for this case series were obtained from MotherToBaby Pregnancy Studies conducted by the Organization of Teratology Information Specialists. MotherToBaby conducts prospective cohort studies of exposures and pregnancy outcomes, drawing subjects from the USA and Canada. Women can enrol in the cohort any time after recognition of the pregnancy, with structured telephone interviews performed at intake, 20 and 32 weeks of gestation. These interviews are designed to collect information about exposures that took place in the 2 years before conception and until delivery. Information on pregnancy outcomes and the health status of the infant, who is followed up to 1 year of age, is obtained from maternal report after delivery and from medical records released by the mother. When possible, dysmorphology examinations are conducted by study physicians to identify clusters of minor anomalies that might constitute a valuable clue in the recognition of a specific pattern of malformation that could include intellectual disability.

All participants provided informed consent. The study was approved by the University of California San Diego Institutional Review Board and complied with the Declaration of Helsinki.

### Exposure

Women who reported use of rituximab within 2 years before conception or for any length of time during pregnancy were eligible for inclusion in the case series. The indication, dose, timing of exposure and route of administration were obtained from a combination of medical records and maternal reports.

Participants were categorized into one of three groups, depending on when they used rituximab relative to conception and their estimated exposure after last infusion, based on the half-life of the drug. Group A included women who reported use of rituximab any time after conception through to delivery. Group B included women who received their last rituximab infusion before conception but were presumed to have some pregnancy exposure, based on a half-life of 20.7 days and an estimated clearance time of 14.8 weeks [1]. Group C included women who reported using rituximab any time from 2 years (104 weeks) until 14.8 weeks before conception.

### Outcomes

The pregnancy outcomes assessed included spontaneous abortion before 20 weeks of gestation, stillbirth (infant demise at or after 20 weeks of gestation), pregnancy complications (pre-eclampsia, gestational hypertension, fever, gestational diabetes, placenta praevia, placental abruption, maternal death, oligohydramnios, bleeding, polyhydramnios, premature rupture of membranes, chorioamnionitis or other infection, HELLP syndrome, incompetent cervix and placenta/cord abnormalities) and premature delivery (<37 weeks of gestation). The infant outcomes assessed included major and minor congenital anomalies, small for gestational age (SGA; <10th percentile for weight at birth) and neonatal complications, including jaundice, meconium aspiration, transient tachypnoea, bradycardia, cyanosis, respiratory problems, resuscitation, hypoglycaemia, jitteriness, hypertonia, hypotonia, temperature regulation issues and neonatal intensive care unit (NICU) admission. An other category, which captured additional neonatal complications not included in the above list, was also evaluated. Serious or opportunistic infections reported within the first year of infant life were also assessed and included pneumonia, sepsis, meningitis, osteomyelitis, bacteraemia, septic arthritis, abscess, mycobacterial infections, invasive fungal infections and *Pneumocystis jirovecii* infection.

A combination of maternal interviews, medical record data as available, and dysmorphology examinations were used to describe exposures and outcomes. When information was discordant, medical record data took precedence over maternal report. When records were unavailable, we deferred to maternal report. Minor congenital anomalies were captured only from a dysmorphology examination that was conducted by a study physician.

### Co-exposures

Additional exposures of interest included suspected or known teratogens used in the first 12 weeks of pregnancy, and tobacco, illicit drugs, psychotropic medications and opioids used any time during pregnancy. Alcohol exposure was classified as a known or suspected teratogen based on dose and frequency, which was defined as at least six drinks per week for >2 weeks during pregnancy or at least three drinks per occasion on at least two occasions during pregnancy [5]. Other co-exposures of interest were biologics, DMARDs and CSs used at any time during pregnancy.

## Results

Twenty-one women who reported use of rituximab within the 2 years before conception through to delivery were enrolled in the MotherToBaby study between 2007 and 2019 and met criteria for inclusion. Two women were lost to follow-up before known outcome.

Of the remaining 19 pregnancies, 15 women reported an indication of RA or JIA, whereas four women used the medication to treat MS. Twelve women (66.7%) reported planning their pregnancies. Mean gestational age at enrolment was 15.3 weeks, with a range of 4.4–26.1 weeks (data not shown). Education level ranged from high school diploma to advanced degree, with the average being some college.

### Timing of exposure

#### Group A (participants 1–3; Table 1)

Two women received their last infusion shortly after conception. The third woman reported use of rituximab through the second trimester, receiving her last infusion at 25 weeks of gestation.

#### Group B (participants 4–6; Table 1)

**Fig. 1:**
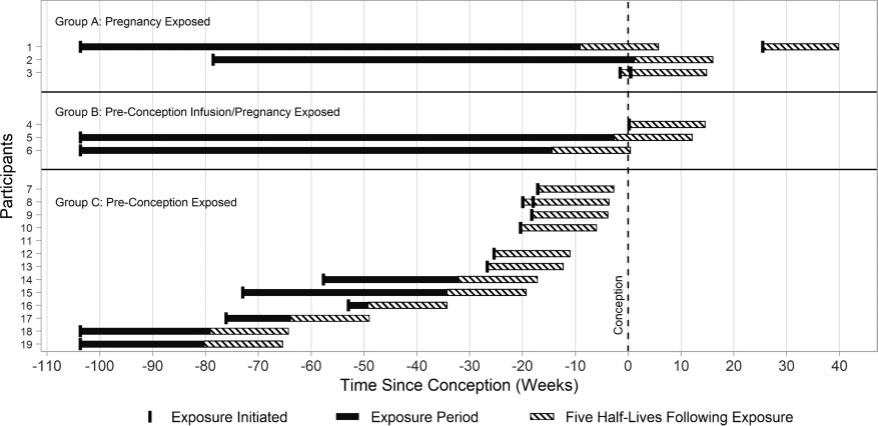
Rituximab exposure relative to conception

Based on the timing of the last dose before conception and estimated clearance time for rituximab, one woman was classified as having gestational exposure until 14.7 weeks of gestation, one until 12.2 weeks of gestation and one until 0.5 weeks of gestation (Fig. 1). Even considering the half-life calculations, it is unlikely that any pregnancies in Group B had significant fetal exposure, given the fact that rituximab is unlikely to cross the placenta before 16 weeks.

#### Group C (participants 7–19; Table 1)

Thirteen women reported receiving their last infusion of rituximab ≥14.8 weeks before conception.

### Pregnancy complications

As shown in Table 1, no spontaneous abortions or stillbirths were reported. One woman in Group A developed pre-eclampsia, and one woman in Group B reported gestational hypertension. In Group C, two women had gestational diabetes, and one woman reported excessive bleeding after a caesarean section. There were two preterm deliveries, both in Group C. One infant in Group A and one infant in Group C were born SGA on birth weight.

**Table 1:** Reported outcomes for participants reporting pre- and postconception rituximab use

Participant number	Rituximab indication	**Dates of rituximab use (weeks from conception)**	Pregnancy complications	Gestational age at delivery (weeks)	Birthweight percentile	Major malformations	Minor malformations	Neonatal complications	Additional exposures of interest during pregnancy
Group A: pregnancy exposure									
1	JIA	−104.0 to −9.0 and 25.2 to 25.2	Pre-eclampsia	38.3	3rd		Examination pending	Jaundice Tachypnoea Respiratory issues Hypoglycaemia Temperature regulation	Sulfamethoxazole Trimethoprim HCQ Prednisone Methylprednisolone
2	RA	−78.9 to 1.3		39.1	58th		Examination pending		HCQ Prednisone
3	RA	−1.9 to −1.9 and 0.1 to 0.1		38.0	22nd		Sparse temporal hair Bulbous nose Bilateral clinodactyly of the fifth finger Broad alveolar ridge Micrognathia Down-turned corners of the mouth Hypotonia	Jaundice Meconium aspiration Cyanosis Respiratory acidosis Resuscitation NICU for 23 days (NAS) Hypotonia Transient pulmonary hypertension Transient thrombocytopenia	Sertraline Oxycodone
Group B: preconception infusion/pregnancy exposure									
4	MS	−0.1 to −0.1		39.0	29th	Multiple (3) haemangiomas	Benign lipoma Long philtrum Aberrant creases	Jaundice	None of interest
5	RA	−104.0 to −2.6	Gestational hypertension	38.9	36th		Vascular malformation		Nitrofurantoin Prednisone
6	MS	−104.0 to −14.3		39.4	37th		Examination pending		None of interest
Group C: preconception exposure (no pregnancy exposure assumed)									
7	RA	−17.4 to −17.4		40.4	79th		Examination completed. None noted by dysmorphologist	Jaundice Respiratory (coarse breath sounds)	Ondansetron
8	RA	−18.3 to −18.3 and −20.3 to −20.3		41.0	47th	Bilateral hip dysplasia	Short palpebral fissure		Prednisone
9	RA	−18.6 to −18.6		39.7	57th		Examination pending		Hyperthermia Sertraline Certolizumab pegol
10	RA	−20.7 to −20.7		40.3	35th		Examination pending	Jaundice	Sulfamethoxazole/trimethoprim
11	RA	−104.0 to −23.3		40.1	8th		Examination pending		Certolizumab pegol SSZ Prednisone
12	RA	Start date unknown to −25.7	Gestational diabetes	36.9 (provider initiated preterm birth)	64th		Bilateral epicanthal folds	Jaundice	Nitrofurantoin Certolizumab pegol
13	RA	−27.0 to −27.0		40.1	57th		Hairwhorl double Plagiocephaly Flat nasal bridge Facial asymmetry Hypertelorism Bilateral clinodactyly of the fifth finger		Certolizumab pegol Betamethasone
14	RA	−58.0 to −32.0	Excessive bleeding (1200 ml) after caesarean section	37.9	19th		Examination pending		Nitrofurantoin Escitalopram HCQ Methylprednisolone
15	RA	−73.3 to −34.0		39.1	43rd		Examination pending	Jaundice	Bupropion Sertraline Certolizumab pegol Prednisone
16	RA/JIA	−53.3 to −49.0		39.0	39th		Examination pending	Jaundice Jitteriness Hypertonia Tremulousness	Ondansetron Amitriptyline Codeine/paracetamol Tocilizumab Prednisone
17	JIA	−76.4 to −63.7		40.0	81st		Plagiocephaly Diastasis recti Uplifted earlobes Bilateral epicanthal folds Micrognathia Capillary haemangioma Non-communicating hydrocoele	Respiratory issues (pneumothoraces) Resuscitation NICU for 1.5 days	Alcohol Cigarettes Marijuana SSZ Tofacitinib HCQ Prednisone
18	MS	−107.57 to −79.0		39.14	17th		Examination pending	Jaundice	Ocrelizumab
19	MS	−104.0 to −80.1	Gestational diabetes	28.3 (PPROM)	54th	Laryngeal cleft	Syndactyly of the toes Mild micrognathia	Jaundice Respiratory difficulties (required ventilation) Resuscitation Digestive issues NICU for 87 days	Bupropion Clonazepam

MS: multiple sclerosis; NAS: neonatal abstinence syndrome; NICU: neonatal intensive care unit; PPROM: preterm premature rupture of the membranes.

### Major congenital anomalies

There were no major congenital anomalies identified in infants born to mothers in Group A. In Group B, one participant with a preconception infusion 0.1 weeks before conception, who had assumed first trimester exposure, delivered an infant with multiple haemangiomas. In Group C, one woman received an infusion 18.3 weeks before conception and had an infant with bilateral hip dysplasia, and one woman received her last infusion 80.1 weeks before conception and her infant was diagnosed with a laryngeal cleft.

### Minor congenital anomalies

Table 1 outlines all the minor malformations that were identified in the nine infants that were examined by a study dysmorphologist. Four of these infants were found to have three or more minor malformations, none of which represented a consistent pattern or cluster of specific features. One of the four, an infant in Group B, also had multiple haemangiomas, which were classified as a major defect. When looking at all the minor malformations identified within the entire group, the findings were diverse. However, similarities with Hoffman syndrome, a genetic condition associated with absence of B cells, were seen.

### Neonatal complications and paediatric infections

Jaundice was seen in 10 of 19 (53%) liveborn infants, and respiratory complications were reported in 5 of 19 (26%) infants. There were no serious or opportunistic infections identified in any infant and no cases of B cell depletion reported. There was one case of transient thrombocytopenia reported in an infant in Group A.

## Discussion

This case series of 19 women adds to the limited data available on the use of rituximab before and during pregnancy. We found no evidence of an excess of adverse pregnancy or infant outcomes in this sample. Specifically, there were three diverse major congenital anomalies (multiple haemangiomas, bilateral hip dysplasia and a laryngeal cleft) identified in liveborn infants, and no specific pattern of minor anomalies in those infants who received the dysmorphology examination. There were no miscarriages or stillbirths. The two preterm deliveries reported in this series were consistent with the background rate of 10% [6] and even lower than the higher rate of preterm delivery often noted for women with RA [7]. The two term infants born SGA in this sample were consistent with the expected rate of ∼10% of infants [8] born SGA in the USA. Other pregnancy complications occurred at or below expected rates for the general population.

Given the known risk of B cell depletion among adults taking rituximab, there is a theoretical concern that the same depletion could occur among fetuses exposed to this medication *in utero*. This was found in 9 of 23 (39%) exposed newborns whose B cell count was measured, in a systematic review of 22 publications by Das *et al.* [9] All nine had normal counts within 6 months of age. Therefore, a careful evaluation of neonatal complications and serious or opportunistic paediatric infections was important to include in this case series in order to assess the postnatal immune function of these infants.

In this series, neonatal complications did not appear to be elevated, and many of the complications reported could be explained by a co-exposure that the woman had during pregnancy. The most common complication reported in our sample was jaundice (10 of 19, or 53%) compared with ∼60% of term babies [10] in the general population. No serious or opportunistic postnatal infections were reported in this series, and there were no cases of B cell depletion noted. The one case of transient thrombocytopenia in Group A is consistent with the population estimate of 1–5% of newborns at birth [11–13]. However, it should be noted that haematological testing for infants in this sample was rarely documented in the medical records received.

Previous publications on exposure to rituximab surrounding pregnancy are limited. A study of 74 pregnancies in Kaiser Permanente patients with MS showed no increase in adverse perinatal outcomes over expected population rates. [14]. The largest dataset available comes from the rituximab global drug safety database maintained by Biogen Idec/Genentech/Roche [2]. Data from 153 pregnancies with known outcome were reported. Of these, 90 resulted in live births, and 61 (68%) were full term. Two of the 90 live births were exclusively exposed in the first trimester of pregnancy. A total of 33 of 153 (21%) ended in miscarriage, and 28 (18%) were terminated electively. One stillbirth occurred owing to an umbilical cord knot. There was one neonatal death at 6 weeks without clear aetiology in a mother who had last taken rituximab 14 months before pregnancy. Concomitant exposure to potentially teratogenic medications occurred in more than half of pregnancies in the database. Although our case series identified one case of thrombocytopenia, 11 neonates (12%) in the pharmacovigilance database had transient haematological abnormalities, including four with B cell depletion, three with lymphopenia, three with thrombocytopenia and one with neutropenia and anaemia. Four neonatal or placental infections were also reported (fever, bronchiolitis, cytomegalovirus hepatitis and chorioamnionitis). Although our study did not tabulate non-serious infections, we found no evidence of serious or opportunistic infections in infants with any timing of rituximab exposure. Two congenital malformations were identified in the pharmacovigilance database: clubfoot in one twin, and cardiac defects (ventricular septal defect, patent foramen ovale and patent ductus arteriosus) in a singleton. These findings are consistent with the 3–5% rate of major birth defects in the general population. Based on the nature of pharmacovigilance reports that are largely retrospective and can be biased towards selective reporting of adverse outcomes, it is difficult to use these data to estimate risk after exposure to rituximab.

In a 2019 abstract, eight adverse outcomes were detailed by Zagorodnikova *et al.* [15] after second and third trimester administration of rituximab for lymphoma (*n* = 6) or SLE (*n* = 2). There were five preterm deliveries (four in women with lymphoma and one in a woman with SLE) and one stillbirth (attributed to intrauterine infection). One infant had atrial and ventricular septal defects. Four liveborn infants had haematological abnormalities, such as neutropenia, lymphocytopenia and thrombocytopenia/anaemia, and one had severe hypoglycaemia. Six placentas were analysed and showed signs of infection. Four children had infections in the neonatal period: cytomegalovirus, necrotizing enterocolitis with pneumonia, bacterial respiratory infection and one unspecified infection. Two children had a mild delay in acquisition of gross motor skills, three had speech delay, and one was diagnosed at 1.7 years with spastic paraplegia. The underlying maternal diseases and concomitant medication use made it difficult to attribute adverse outcomes to rituximab alone. In this small exposure series reported in abstract only, the numbers of preterm deliveries, infant and placental infections and haematological abnormalities exceeded our findings. However, there were only three women in our sample with any rituximab dose given in pregnancy and only one with second or third trimester exposure.

Minor anomalies are subtle differences that are in and of themselves cosmetically or medically insignificant and are not associated with functional impairment. However, a pattern of three or more specific minor anomalies is a potential red flag for the presence of an associated major malformation, which can be occult, such as a defect in brain development [16, 17]. Four of the nine children who received a dysmorphology examination in this case series were found to have three or more minor malformations, but no consistent pattern was identified, and only one of these children was also found to have a major birth defect (multiple haemangiomas).

Although no specific cluster of minor anomalies was identified in this series, a few of the isolated minor malformations that were identified are similar to those seen in individuals with Hoffman syndrome, a genetic disorder associated with absence of B cells [18]. These include two instances of bilateral clinodactyly of the fifth finger, two infants with bilateral epicanthal folds, and three infants with micrognathia. Whether or not these findings are associated with underlying B cell depletion in the children is unknown, because the medical records received did not indicate performance of laboratory testing for B cell status among any of the children who had a dysmorphology examination in our case series. Only one woman reported having lymphocyte subset levels (assessing T, B and NK cells) assessed on her infant, which were reported to be within normal limits. Although the presence of any of these minor anomalies alone is not indicative of concern for infants born to women who have been treated with rituximab before or during pregnancy, this merits consideration in future research. Health-care providers who are overseeing care for infants with prenatal exposure to rituximab should consider ordering laboratory work that assesses B cell status, in an attempt to provide a more complete picture of immune function. Additionally, further research on this topic should focus on long-term follow-up of exposed infants with respect to immune function.

### Strengths and limitations

This study has several limitations and strengths. One limitation is that we had small numbers of women with exposure in each of the three defined time windows. A second limitation is that we estimated timing of exposure based on a half-life derived from adults being treated for RA, because most women in the sample were receiving rituximab for that indication [1]. However, the half-life of the drug could have varied in women with different indications, or among those who received higher or more frequent doses, potentially resulting in misclassification of exposure timing. A third limitation is that complete data from all requested medical records (obstetric, hospital, paediatric and specialist) were available for only 13 of the 19 participants. The remaining six had a partial set of records, which meant that maternal report was the only source of information for some outcomes.

A major strength of this analysis is the inclusion of minor malformations assessed by trained dysmorphologists in a subset of infants, which are rarely reported in the literature. A second strength in this analysis is the inclusion of women who are being treated for RA, JIA and MS. Much of the existing literature on rituximab use in pregnancy focuses on women who are being treated for indications such as B cell, CD20-positive non-Hodgkin lymphoma. These pregnancies are often complicated by compromised maternal health status and concomitant use of teratogenic medications (such as MTX or chemotherapy), which might confound analyses and thus limit generalization to other conditions being treated with rituximab in clinical practice.

### Conclusion

Pregnancy complicated by an autoimmune condition is optimally accomplished with anticipatory guidance and a partnership created between the patient, her specialist and her obstetrician. Discussions should be undertaken to assess medication risks, disease severity and access to specialty care should an exacerbation of the condition occur.

No evidence of any excess of serious adverse outcomes was seen in this small sample of children born to women who took rituximab before or during pregnancy. Women who are deciding whether to continue use of rituximab in pregnancy should understand that the available data are still insufficient to document safety and recommend the use of rituximab during pregnancy, but they should be aware that no patterns of birth defects, pregnancy complications or negative infant health outcomes were observed in the small case series presented here.

## Acknowledgements

This work was performed with support from Diana Johnson, Norma Kelly and Gordon Honerkamp-Smith. Lori Broderick MD, PhD was instrumental in helping us to evaluate the lymphocyte subset level reported by one participant in this case series. We would also like to thank personally the MotherToBaby study participants, without whom this work would not be possible, and the physicians who examined these children, including Kenneth Lyons Jones, Margaret Adam, Miguel Del Campo, Leah Burke and Keith Vaux.

*Funding:* No specific funding was received from any bodies in the public, commercial or not-for-profit sectors to carry out the work described in this article.

*Disclosure statement:* C.C. receives research funding from the following industry sponsors and foundation: Amgen Inc.; AstraZeneca; Celgene; GlaxoSmithKline; Janssen Pharmaceuticals; Pfizer, Inc.; Regeneron; Hoffman La-Roche-Genentech; Genzyme Sanofi-Aventis; Takeda Pharmaceutical Company Limited; Sanofi; UCB Pharma, USA; Sun Pharma Global FZE and the Gerber Foundation. The other authors have declared no conflicts of interest.

## Data availability statement

Data are available upon reasonable request by any qualified researchers who engage in rigorous, independent scientific research, and will be provided following review and approval of a research proposal and Statistical Analysis Plan (SAP) and execution of a Data Sharing Agreement (DSA). All data relevant to the study are included in the article.
